# Late Holocene wetland transgression and 500 years of vegetation and fire variability in the semi-arid Amboseli landscape, southern Kenya

**DOI:** 10.1007/s13280-018-1014-2

**Published:** 2018-02-03

**Authors:** Esther N. Githumbi, Colin J. Courtney Mustaphi, Kevin J. Yun, Veronica Muiruri, Stephen M. Rucina, Rob Marchant

**Affiliations:** 1 York Institute for Tropical Ecosystems, Environment Department, University of York, Heslington, York, YO10 5NG UK; 2 Department of Archaeology and Ancient History, Uppsala Universitet, P.O. Box 256, 751 05 Uppsala, Sweden; 3 Department of Biosciences, School of Biological and Biomedical Sciences, Durham University, Stockton Road, Durham, DH1 3LE UK; 4 Department of Earth Sciences, Palynology and Palaeobotany Section, National Museums of Kenya, P.O BOX 45166 00100, Nairobi, Kenya; 5 Hong Kong Baptist University, Hong Kong, China

**Keywords:** Amboseli, Charcoal, Environmental history, Pollen, Savanna, Swamps

## Abstract

The semi-arid Amboseli landscape, southern Kenya, is characterised by intermittent groundwater-fed wetlands that form sedimentary geoarchives recording past ecosystem changes. We present a 5000-year environmental history of a radiocarbon dated sediment core from Esambu Swamp adjacent to Amboseli National Park. Although radiocarbon dates suggest an unconformity or sedimentary gap that spans between 3800 and 500 cal year BP, the record provides a unique insight into the long-term ecosystem history and wetland processes, particularly the past 500 years. Climatic shifts, fire activity and recent anthropogenic activity drive changes in ecosystem composition. Prior to 3800 cal year BP the pollen data suggest semi-arid savanna ecosystem persisted near the wetland. The wetland transgressed at some time between 3800 and 500 cal year BP and it is difficult to constrain this timing further, and palustrine peaty sediments have accumulated since 400 cal year BP. Increased abundance of Afromontane forest taxa from adjacent highlands of Kilimanjaro and the Chyulu Hills and local arboreal taxa reflect changes in regional moisture budgets. Particularly transformative changes occurred in the last five centuries, associated with increased local biomass burning coeval with the arrival of Maa-speaking pastoralists and intensification of the ivory trade. Cereal crops occurred consistently from around 300 cal year BP, indicative of further anthropogenic activity. The study provides unique insight in Amboseli ecosystem history and the link between ecosystem drivers of change. Such long-term perspectives are crucial for future climate change and associated livelihood impacts, so that suitable responses to ensure sustainable management practices can be developed in an important conservation landscape.

**Electronic supplementary material:**

The online version of this article (10.1007/s13280-018-1014-2) contains supplementary material, which is available to authorized users.

## Introduction

Rapid ecosystem changes, particularly during the recent past, are primarily attributed to anthropogenic modifications that are superimposed on long-term climatic and landscape-scale changes (Young 2014). East African savannas support large human (Lane 2013) and herbivore (Sarkar 2006) populations and are currently undergoing rapid development and pressure on water resources (Bond and Midgley 2000; Thenya 2001). These changes are leading to wildlife habitat degradation and fragmentation (Moses et al. 2016), biodiversity loss (Western and Maitumo 2004; Muchiru et al. 2009), and other stressors surrounding the community conservation nexus. To understand, and uncouple, how these abiotic and biotic factors interact it is crucial to have a long-term perspective on ecosystem change that can be used to understand how entangled interactions between the environment, ecosystems and humans influence the current state of the ecosystem and possible future trajectories (Marchant and Lane 2014).

**Fig. 1:**
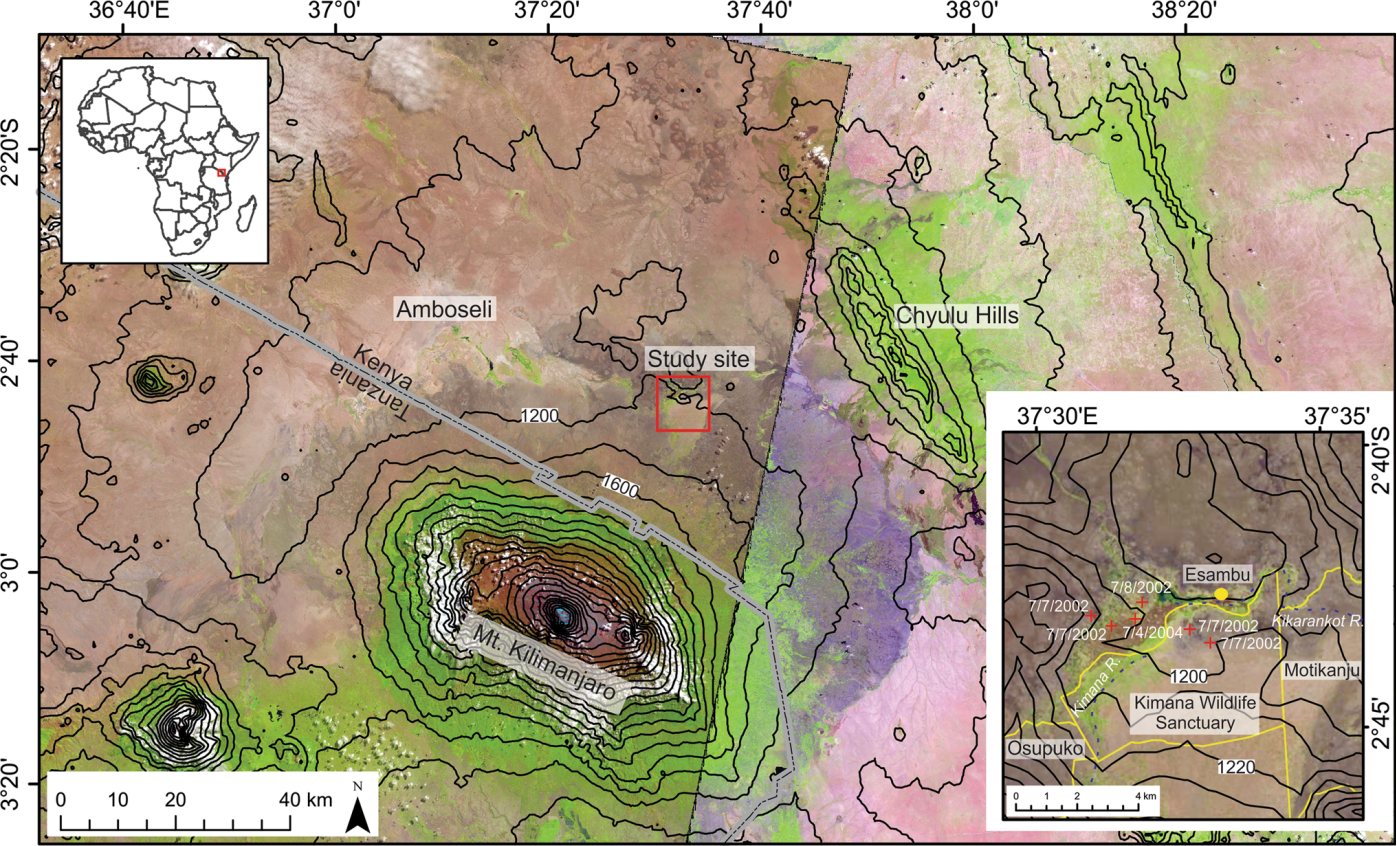
Location map of the study region in equatorial eastern Africa (top left inset). Esambu coring site (Inset, 10 m elevation contours) within the greater Lielerai-Kimana Swamp and the community conservancies (yellow delimitations) that create a corridor through the wetlands westward toward Amboseli National Park (main map, 200 m elevation contours). The core was collected at a swamp (yellow circle) within the Esambu Irrigation Scheme area. Red ‘+’ symbols indicate historical fire hotspots within the MODIS FIRMS MOD14/MYD14 satellite-derived product (date format month/day/year). Basemap LandSAT images LC81680622014194LGN00 (left side and inset) LC81670622015014LGN00 (right side)

The Amboseli wetlands of southern Kenya sustain perennially high local water tables supporting grazing lawns and long grasses, and enable tropical peat accumulation at shallow pools, which persist through drought periods. These wetlands recharge from orographic precipitation falling on Kilimanjaro and the Chyulu Hills. They provide an important source of water, wildlife refuge, and form a series of ‘grazing stepping stones’ for animal migrations between Amboseli National Park and neighbouring Tsavo and Chyulu Hills National Parks (Fig. 1). The more ephemerally moist fringes of the wetlands and the surrounding ecosystems are sensitive to hydrologic and climatic variability, and are highly responsive to changing human land use practices and land cover modifications (Casanova and Powling 2014).

Reconstructing the vegetation, climate and fire dynamics is of particular interest as the Amboseli landscape supports migratory wildlife that move between the Kenyan and Tanzanian border. An understanding of the response of the remaining Amboseli wetlands to changes in driving factors such as climate change, wildlife use, fire and anthropogenic factors over time is necessary to inform sustainable wetland management of the wetlands that are important dry season grazing refuges for wildlife. There have been relatively few palaeoecological studies in southern Kenya due to the extensive coverage of semi-arid savanna ecosystems, woodland and scrub vegetation with limited suitable sedimentary basins providing few localised records (Rucina et al. 2010). We present pollen, non-pollen palynomorph and macroscopic charcoal data providing insights into the evolution of the wetland and the interaction of fire, vegetation and climate within the Amboseli ecosystem.

## Study region

### Climate and geologic setting

The Amboseli landscape (Fig. 1) is characterised by five main components: gently undulating basement plains on deep, acidic, well-drained soils; palaeolacustrine basin and swamps; basal slopes of Kilimanjaro with deep, well-drained neutral soils; volcanic landscapes with abundant small to block sized volcanic cones, reddish brown scoria pyroclasts and shallower soils that support semi-arid woodland and scrub; and denser woodland savanna on young basaltic parent material toward Chyulu Hills.

Vegetation in the region is characterised by sparse shrubland savanna and *Acacia*-*Commiphora* dominated dry woodland-lowland savanna. Throughout this paper, we use *Acacia* to describe African acacias of *Vachellia* and *Senegalia* genera. Crosscutting these landscapes are riparian wooded areas following semi-permanent and seasonal channels draining northward from Kilimanjaro and then eastward to converge with the Galana River catchment to Tsavo.

**Fig. 2:**
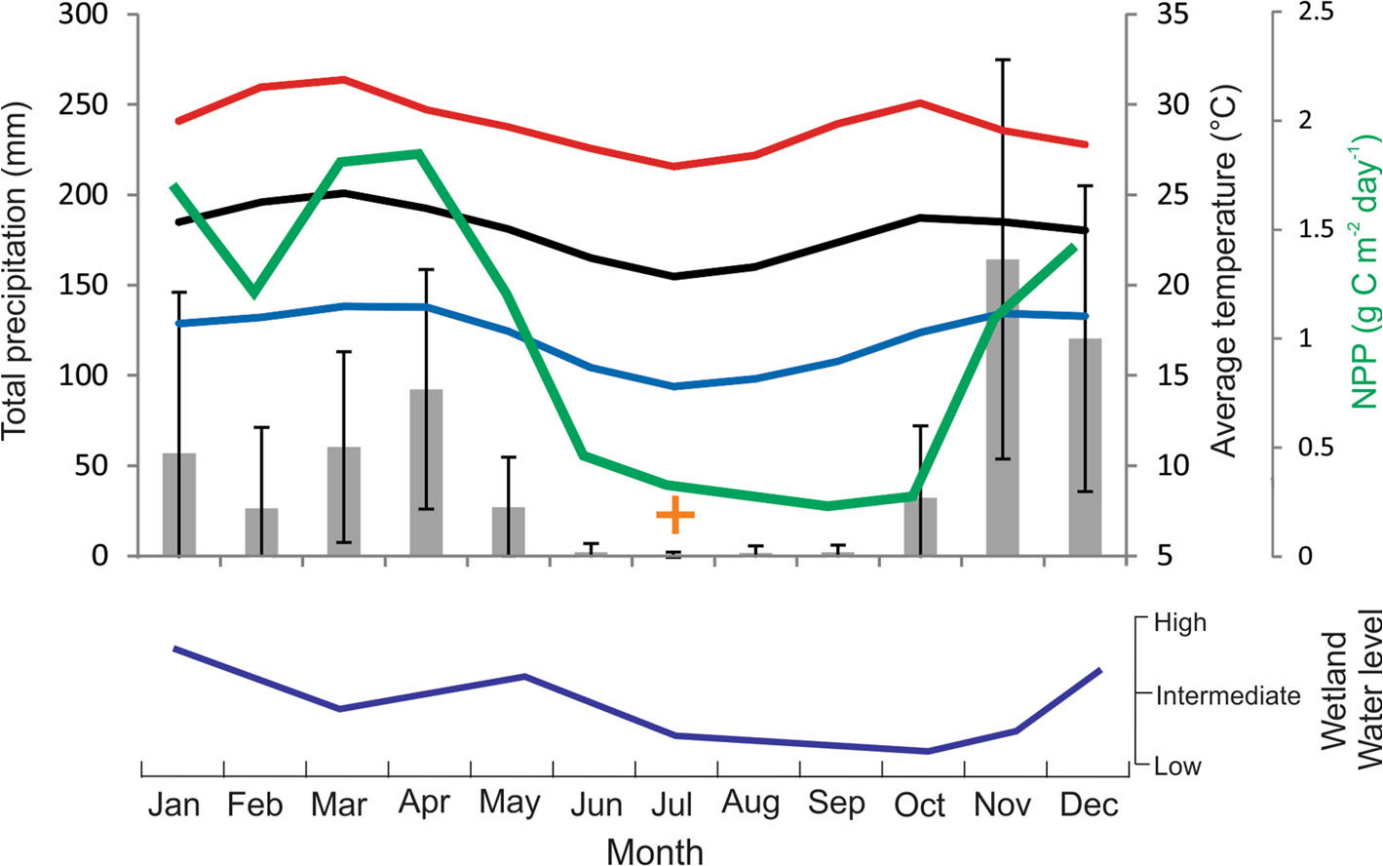
Mean monthly total precipitation (grey bars) and 1*σ* standard deviations (whiskers). Mean monthly (black line), maximum (red line) and minimum (blue line) temperatures from 1979 to 2009 recorded at Makindu meteorological station (Kenya Meteorological Department), which is ~ 60 km west of the study site, and monthly net primary productivity (NPP, green line, MOD17A2 for 2010 at 0.25 degree resolution, average of four grid cells 2.625–2.875°S, 37.375–37.625°E). The orange ‘+’ symbol represents July peak fire detections within the satellite-derived fire hot spots detected (MODIS FIRMS MOD14/MYD14 product 2001–2013; 5 fires in July 2002 and 1 fire in July 2004). Lower graph shows qualitative representation of the monthly water level fluctuations in eastern Amboseli area swamplands with a 1–2 month lag response following the bimodal rainy seasons as described in 2014 by members of the Namelok Irrigation Scheme

The climate of Amboseli is characterised by high average annual temperatures (23 °C) with little annual variability and a bimodal precipitation regime with short rains falling from October to December and long rains falling from March to May (Fig. 2). November and April are the two wettest months while July is the driest. Average annual precipitation is 586 mm year^−1^ with considerable inter-annual variation. The locals observe the change in the Esambu water table and can describe the general pattern of change across the year (Fig. 2). Locally, the precipitation distribution is moderated by the topography of Kilimanjaro; precipitation falling on Kilimanjaro recharges groundwater flows into the lowlands of Amboseli (Sarkar 2006). Surface runoff percolates into the basaltic bedrock with the resulting groundwater flowing towards the Amboseli Basin that maintains the wetlands via perennial springs during dry periods. To the east of the area is Lake Amboseli (1125 m a.s.l.) that supports standing water during more mesic wet seasons although it has remained relatively dry since the 1960s (Directorate of Overseas Surveys 1963a, b; Williams 1972).

### Recent land use changes across the Amboseli area

Land cover changes, notably between open savanna and woody savanna, have been quite common across Africa in response to indirect and direct human activities and climatic changes (Gillson and Marchant 2014). In North Pare, Tanzania, an increase in tree cover after the 19th century was noted after British colonial land use policies encouraged agroforestry to meet timber demand leading to the increased planting of exotic species such as *Eucalyptus* (Gillson 2004). In Amboseli, control of the movement of elephants has been proven to drive woodland density (Western and Maitumo 2004) with high elephant numbers causing a loss in woodland. There have been rapid ecosystem changes to the Amboseli area over the past 100 years caused by animal disease outbreaks, agricultural expansion, watercourse diversion, reduction of tree cover, and water extraction from the wetlands outside of protected areas (MEMR 2012). There is increasing concern regarding the management of the area due to competition for water resources by humans and wildlife, global temperature increases, the notable decrease in rainfall on Kilimanjaro that has reduced ~ 36% since 1910 and the extensive loss of cloud forests (Hemp 2005). The hydrological loss from the destruction of montane and cloud forests on Kilimanjaro is greater than that of the glaciers retreat (Hemp 2005). Recently, the wider Amboseli landscape experienced major droughts in 1984, 1994 and 2008–2009 that have emphasised the need for multi-stakeholder conservation and management of wetlands due to rapid increases in human population and associated land use pressures (MEMR 2012). A 10-year management plan was developed through participation between Amboseli Basin stakeholders that builds on the previous management plan (1991–1996) (Kenya Wildlife Service 2008).

### Study site

**Fig. 3:**
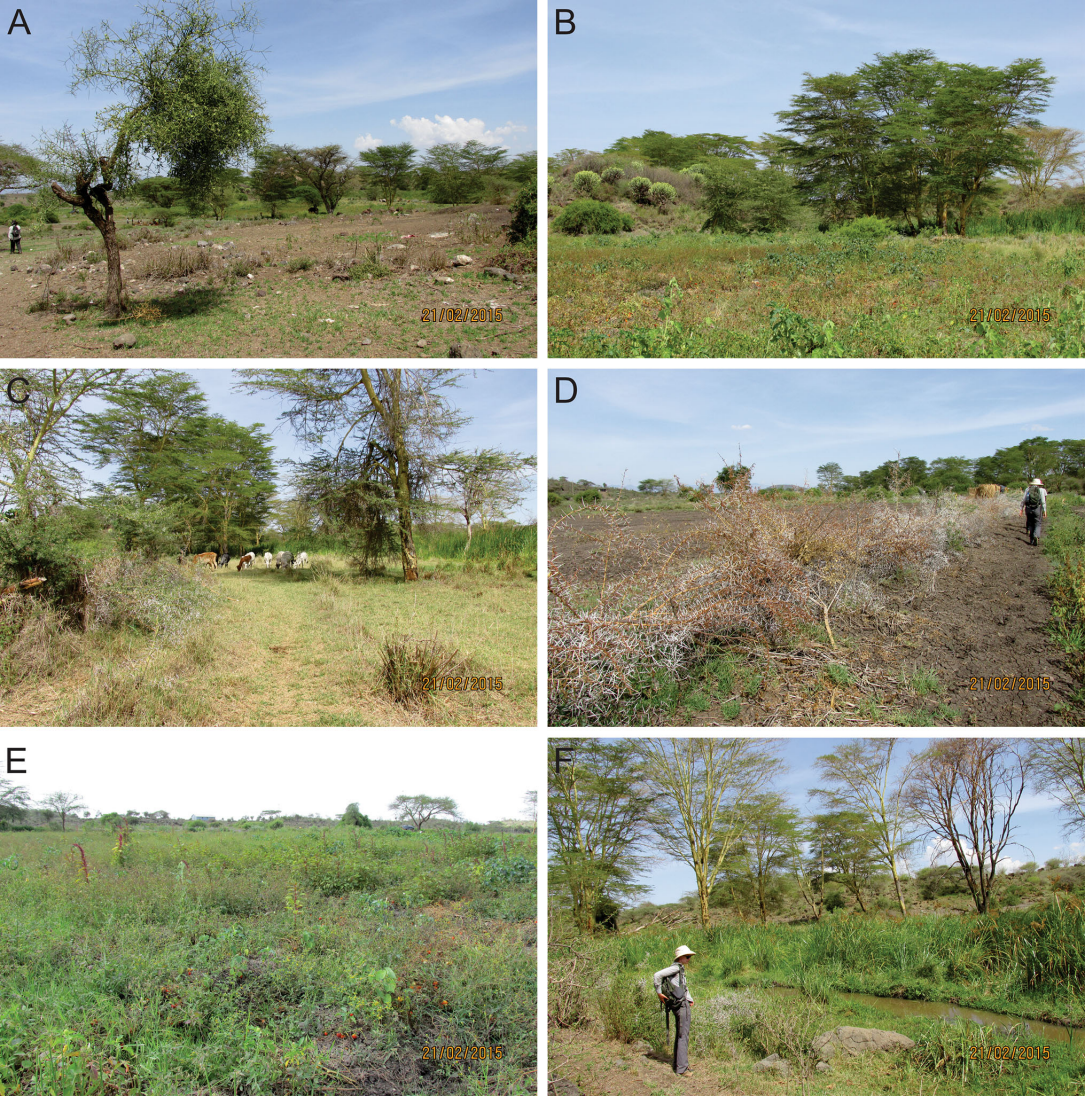
Representative ecosystem photographs **a** common mix of *Commiphora* and *Acacia* trees, grasses and barren patches surrounding the wetland ecosystems at the drylands-savannah interface. **b** Typical wooded savannah and a volcanic mound at left hosting xerophylic taxa, such as young *Euphorbia*. **c** Cattle grazing within the savannah around the swamp. **d** An acacia fence used to control grazing access to the moister swampy regions. **e** Tomato farming within the ruderal herbaceous, woody and grass taxa surrounding the swamp. **f** The emergent aquatic plants of the hydric sediments within the swamp, dominated by Cyperaceae. Photographs: Esther Githumbi

Indoinyo Esambu Swamp (1191 m a.s.l.) is a ~ 0.4 km^2^ wetland associated with the Kikarankot River that supplies water to an area of ~ 12 000 km^2^ with a population of ~ 100 000 people (Directorate of Overseas Surveys 1963a; Wetlands International 2009). The vegetation cover within Esambu Swamp is dominated by Cyperaceae-Poaceae with *Cyperus rotundus*, *C. papyrus* and *Typha* growing in the wetland and *Acacia xanthophloea* dominating the riverine arboreal taxa. Large areas of the wetland have been cultivated and the semi-arid woodland, beyond the margins of the wetland, is sparsely vegetated with co-dominant *Acacia*, *Balanites*, *Commiphora* and *Euphorbia* (Fig. 3).

Pastoralist populations with historical formal and informal land tenure have recently reorganised into more sedentary group ranches whereby some groups and individuals maintain pastoral livelihoods on an increasingly fragmented socio-ecological landscape. People have migrated into the region and the increased population have placed additional demand on the Amboseli wetlands (Sarkar 2006). Land cover and land uses include commercial agriculture, particularly expansive in and around the wetlands, pastoralism across the landscape, rural urbanisation, and various protected areas including National Parks, community and private game reserves.

## Materials and methods

### Core collection

A 247.5-cm core was collected in 2009 at − 2.711913, 37.554357 (1191 m a.sl.) using a Russian corer with a 5-cm-diameter, 50-cm-long, hemi-cylindrical collection chamber from proximal, overlapping (~ 10 cm), parallel boreholes through 10 cm of standing water. Cores sections were wrapped in aluminium foil and transferred to split plastic PVC pipes for transport and refrigeration at the Palynology and Palaeobotany Section, National Museums of Kenya (NMK), Nairobi. Subsamples were extracted for pollen analysis at NMK and subsamples sent to the University of York, UK, for charcoal analysis.

### Geochronology and age-depth modelling

One *Acacia* sp. wood fragment and five bulk organic sediment subsamples were accelerator mass spectroscopy (AMS) radiocarbon dated. Samples sent to the ^14^CHRONO laboratory (Queen’s University Belfast, Northern Ireland) were acid wash pre-treated prior to combustion to CO_2_, graphitization and AMS using a NEC 0.5 MV compact accelerator (Reimer 2015). Samples sent to Direct AMS (Bothell, WA, USA) were acid–base–acid washed, combusted, graphitized and measured using a National Electrostatics Corporation (NEC) 1.5 SDH Compact Pelletron 500 kV accelerator mass spectrometer. Reported dates were corrected for instrumental isotopic fractionation ^13^C values by the respective laboratories. Radiocarbon dates were calibrated using the IntCal13 curve (Reimer et al. 2013) and used to build a linear interpolated age-depth model using the *R* script *Clam* version 2.2 (Blaauw 2010) for the upper palustrine peaty sediments. Lower sediments were not age-depth modelled and the radiocarbon dates were used to examine the maximal time interval that represented wetland transgression, a sediment gap and potential unconformities. This information provides palaeoenvironmental context for the history of the site. Henceforth, we refer to all calibrated ages as years before present (year BP), CE 1950 (Common Era).

### Pollen and palynomorph analysis

Sediment cores were subsampled for 1 cm^3^ of wet sediment taken from 100 depths at 2.5-cm intervals down the core and pollen and non-pollen palynomorphs (NPP) were isolated using sequential chemical digestion (Fægri and Iversen 1989). Pollen identification was performed on 70 levels using ×400 and ×1000 magnification to a minimum count of 350 grains. The other 30 pollen samples were left out due to low pollen numbers (> 350). Pollen identifications were made with the aid of the pollen reference collection at the Palynology and Palaeobotany Section, NMK, Nairobi. Relative abundances were calculated from the terrestrial pollen sum and aquatic taxa relative abundances were calculated from the aquatic taxa sum of Cyperaceae, *Hydrocotyle*, *Myriophyllum*, *Nymphaea* and *Typha*. NPP, including fungal spores, were identified from 100 levels with the aid of a taxonomic key (van Geel et al. 2011) and the relative abundances of spore types were calculated using the total NPP sum per sample.

### Macroscopic charcoal analysis

A total of 125 subsamples of 1 cm^3^ were collected at intervals of between 1 and 5 cm. To each subsample, sodium hexametaphosphate and distilled water were added to disaggregate the samples and aid in separation of organic material and clays. Samples were then washed through a 125-μm sieve and the retained charcoal particles tallied under a Zeiss Stemi 2000-C stereomicroscope between 10 and ×50 magnification and charcoal concentrations were converted to charcoal concentrations (pieces cm^−3^ of wet sediment) and charcoal accumulation rates (CHAR, pieces cm^−2^ year^1^).

### Data analyses

Pollen and NPP taxa relative abundances were square-root transformed and CHAR from the same 70 down-core levels were log transformed and included in a principal component analysis (PCA) to examine the correlation structure of the data. Pollen and spore assemblage zones were each identified through constrained hierarchical cluster analysis (CONISS) with the zones being tested for significance using a broken stick test of the sum of squares above the default cut-off implemented with R scripts (R Development Core Team 2015). Pollen taxa were grouped into Afromontane, trees, shrubs, herbs, grasses and aquatics according to their dominant structure at the taxonomic resolution possible through pollen analysis (Table S1).

### Illustrative landscape model

The results from the pollen, spore and charcoal analysis, combined with current observations of the wetland landscape, were used to develop a system-wide visual representation of the key components of the wetland and surrounding ecosystem. CONISS zones derived from the pollen spectra defined the time steps but dates have been rounded for graphical representation. Both planar, schematic and cross-sectional views supplemented with example photographs, were created and used to represent the changes in the system over the late Holocene. The relative importance of varying and interacting spatial controls on fire regimes at centennial to millennial temporal scales is represented using a fire regime triangle diagram (Whitlock et al. 2010). Although not set at an absolute scale, the areal extent of the different land use and land cover components portray the relative dominance of the plant group abundance sums from the wetland and the surrounding ecosystems.

## Results

### Stratigraphy and geochronology

**Fig. 4:**
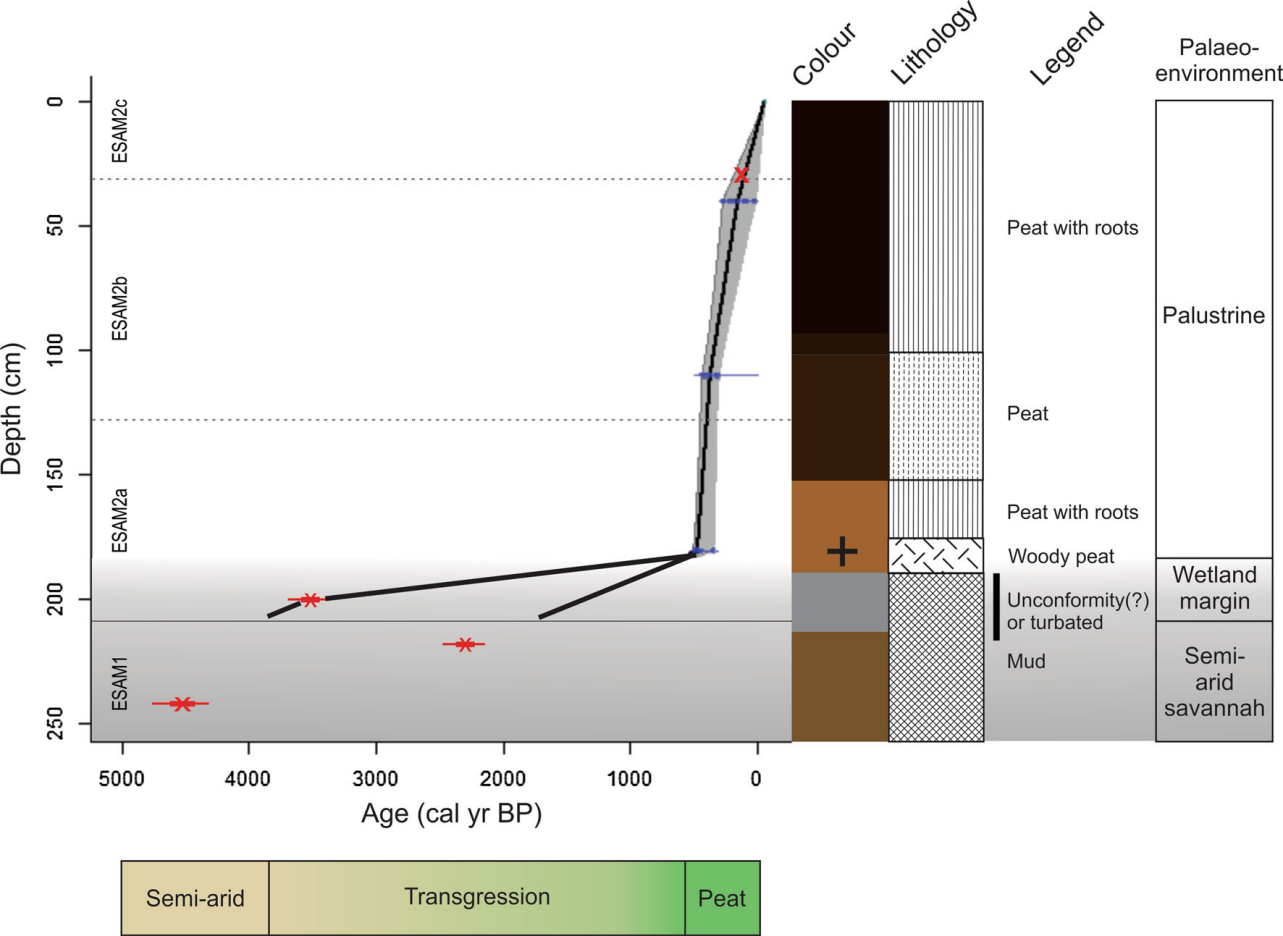
Linear interpolation age-depth model of the core collected from Esambu Swamp. The 95% confidence envelope is shown in grey and ranges span from 4 to 263 year. (average 146 year). A total of 148 iteration models were removed due to age-depth reversals and the weighted mean (black line) of 852 model iterations were used for the final model (− log goodness-of-fit = 16.83). The top red ‘X’ symbol is the location of the first *Zea mays* pollen sample, the next three blue dots are the three radiocarbon dates that are modelled while the bottom three red dots are the radiocarbon dates not modelled. Lithology description with legend description at right side of diagram. The deeper sediment zone with higher chronological uncertainty is shaded in grey with the zone of a potential sedimentary unconformity, turbated sediments of gap is shown with black bar—this separates the lower material that we interpret as an older unit and did not age-depth model due to uncertainty, and an upper unit of undisturbed wetland sediments that have been age-depth modelled. General palaeoenvironmental interpretation of the sediments at far right that is congruent with the ecohydrological schematic at bottom (sandy beige to green)

The 247.5-cm core is characterised by dark brown silty peat from the surface to 120 cm; this changes into grey-brown sediment from 120 to 180 cm, from 180 to 210.5 cm the sediment is a light grey clay, which grades into compact brown clay to the bottom (247.5 cm). Radiocarbon dates show that the sediment record spans from 4974 cal year BP to the present (Table 1, Fig. 4). Linear interpolation age-depth modelling of the uppermost age determinations provided age estimates for the peat section (Fig. 4). The lower three radiocarbon dates contained one reversed date and were treated with large chronological uncertainty in our interpretation and, thus, were not age-depth modelled. The deepest reliable date was at 180.5–186 cm from a piece of *Acacia* sp. wood (Fig. S1). We explored multiple age-depth models iteratively ignoring or including the deeper radiocarbon dates and decided to maintain an age-depth model of the upper reliable dates. After careful examination of the geochronology, we interpret three palaeoenvironmental phases to the sediment profile and use the radiocarbon dates to constrain the probable time interval related to each.

**Table 1:** Geochronological determinations of the Esambu palustrine sediment core

Sample ID	Depth (cm)	^14^C age	1*σ* error (± years)	F^14^C ± 1*σ* error	Sample material
Top	0				Collected in 2009
UBA-27555	40–41	146	23	0.9820 ± 0.0028	Bulk sediment
UBA-27556	110–11	296	24	0.9639 ± 0.0028	Bulk sediment
UBA-26124	180.5–186	394	24	0.9522 ± 0.0029	Acacia wood
D-AMS 009665	200–201	3299	29	0.6632 ± 0.0024	Bulk sediment*
D-AMS 009666	218–219	2296	29	0.7514 ± 0.0027	Bulk sediment*
UBA-26123	242–244	4001	27	0.6077 ± 0.0021	Bulk sediment*
Bottom	247.5				Coring ceased

Thin section images of sample UBA-26124 African acacia (*Vachellia* sp.) wood fragments are presented in Fig. S1

*Dates not used to interpolate ages

The deepest fine-grained sediments accumulated in a semi-arid savanna ecosystem likely proximal to a wetland from 5000 to approximately 3800 cal year BP. Between 3800 and 500–400 cal year BP the proximal wetland of the Kikarankot River expanded and transgressed the coring site. The data do not permit an examination of the rate of this change or the frequency if there were more than a single transgression. The sediments in this marginal wetland phase may have produced radiocarbon age reversals due to erosion, non-deposition, or bioturbation or physical turbation. An erosional origin resulting from the transition from dominantly semi-arid conditions to increased moisture, or a depositional origin caused by increased aridity with subsequent cessation of sediment accumulation. Due to these uncertainties, we interpret this phase as a marginal wetland transgression leading to the current phase of palustrine peaty sediments that accumulated from 400 cal year BP to present (Fig. 4). Ages prior to 400 cal year BP are estimates based on the radiocarbon ages and potential age-depth models, but uncertainty is lowest from 400 cal year BP to present.

### Pollen and NPP assemblage summaries

**Fig. 5:**
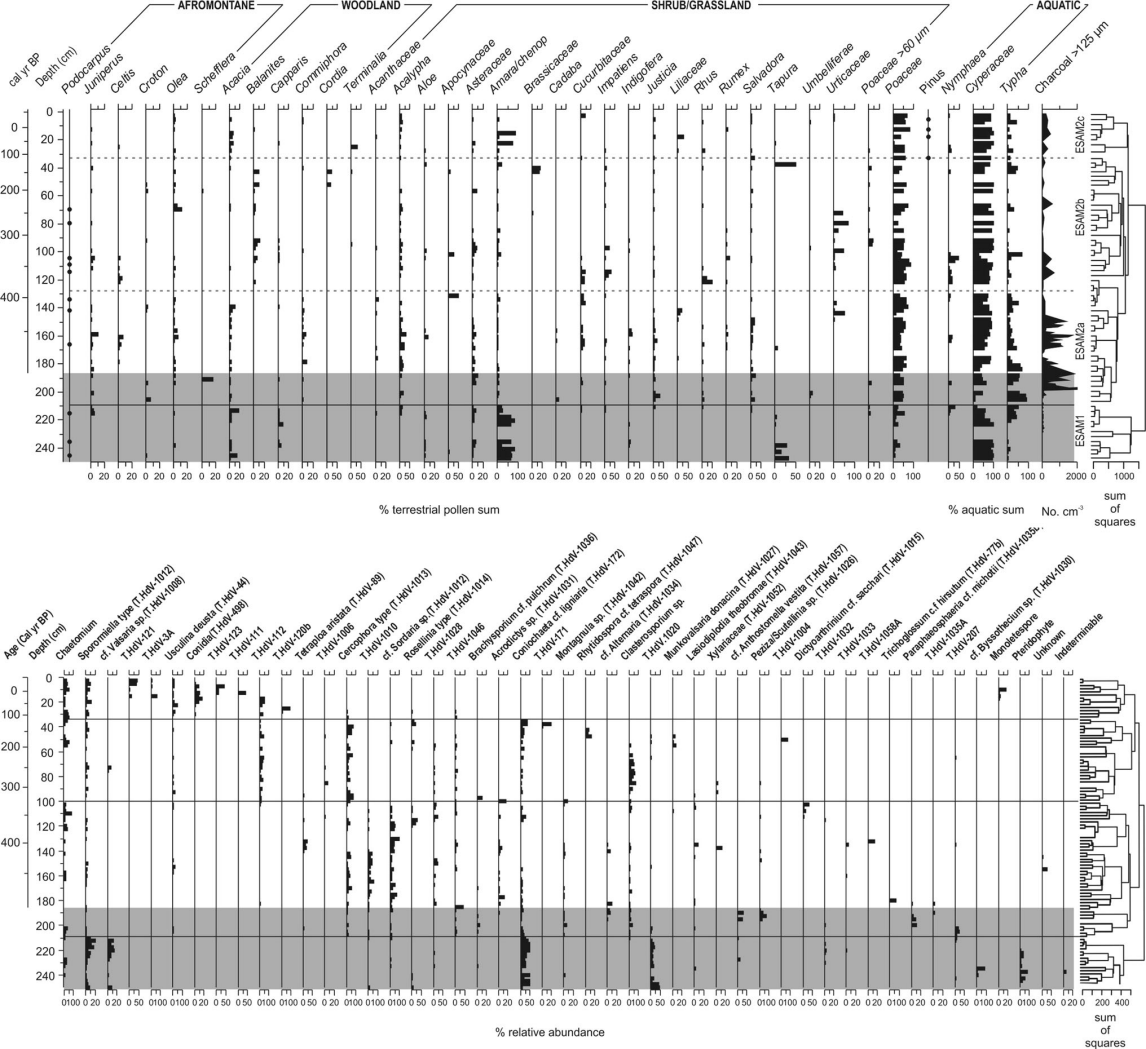
(Top) Relative abundances of selected pollen taxa of terrestrial pollen sum from Esambu presented by depth in the stratigraphy and taxa grouped into plant functional types. Macroscopic charcoal concentration (> 125 µm), unknown and indeterminable taxa not included in the pollen sum nor the CONISS calculation. Two significant CONISS zones separated by a solid grey line, insignificant breaks shown by the dotted grey line. (Bottom) Relative abundances of spore types (van Geel et al. 2011) from Esambu presented by depth in the stratigraphy. Four significant CONISS zones separated by a solid grey line

A total of 93 pollen taxa and 74 NPP types were identified throughout the core. Two pollen zones delineated by CONISS were statistically significant, separating the deeper sediments of ESAM1 representing the semi-arid woodland taxa from ESAM2 showing an expanded wetland and increased sedimentation rates (Fig. 5). Pollen zone ESAM2 was further divided by insignificant CONISS breaks and visual differences observable in the pollen spectra (ESAM2a–c). CONISS zones in the NPP data showed very similar significant transitions in assemblages concomitant with pollen assemblages with the exception of ESAM2a–ESAM2b occurring 50 years later in the NPP assemblages (Fig. 5).

#### Pollen zone ESAM 1: 247.5–210.5 cm

Amaranthaceae-Chenopodiaceae and Cyperaceae dominate this zone, with *Acacia*, *Aloe,* Asteraceae, *Capparis*, Poaceae and *Tapura* consistently present. *Acalypha* and *Juniperus* appear towards the top of the zone (Fig. 5). Eighteen spore types are identified in this zone and the charcoal concentration and accumulation rate is relatively low.

#### Pollen zone ESAM 2a: 210.5–131.5 cm

Cyperaceae, Poaceae, *Salvadora* and *Typha* dominate this zone. Diverse arboreal taxa *Celtis, Commiphora, Croton, Juniperus*, *Olea*, *Podocarpus* and *Schefflera* are also present in this zone. 32 spore types were identified in this zone, and the charcoal concentration and accumulation rate rapidly increase at this level having several charcoal peaks (Fig. 5).

#### Pollen zone ESAM 2b: 131.5–27.5 cm

Cyperaceae, Poaceae and *Typha* dominate this zone. *Acalypha*, Asteraceae, *Impatiens* and *Rhus* have a varied presence throughout the zone. *Balanites* and *Celtis* appear at 330 cal year BP before disappearing at 200 cal year BP (Fig. 5) from this zone. 27 spore types were identified in this zone. The charcoal concentration and accumulation rate are lower than the previous zone with only two peaks in this zone.

#### Pollen zone ESAM 2c: 27.5–2.5 cm

Cyperaceae, Poaceae and *Typha* dominate this zone. Amaranthaceae/Chenopodiaceae, *Salvadora* and Poaceae grains (> 60 µm) appear consistently throughout this zone with *Pinus* appearing at four depths since 110 cal year BP. *Juniperus*, *Celtis* and *Olea* are the only Afromontane species that in this zone. 17 spore types are identified in this zone, with *Sporormiella* occurring consistently (Figs. 5, S2). This zone has very low charcoal concentration levels.

### Landscape interpretation

**Fig. 6:**
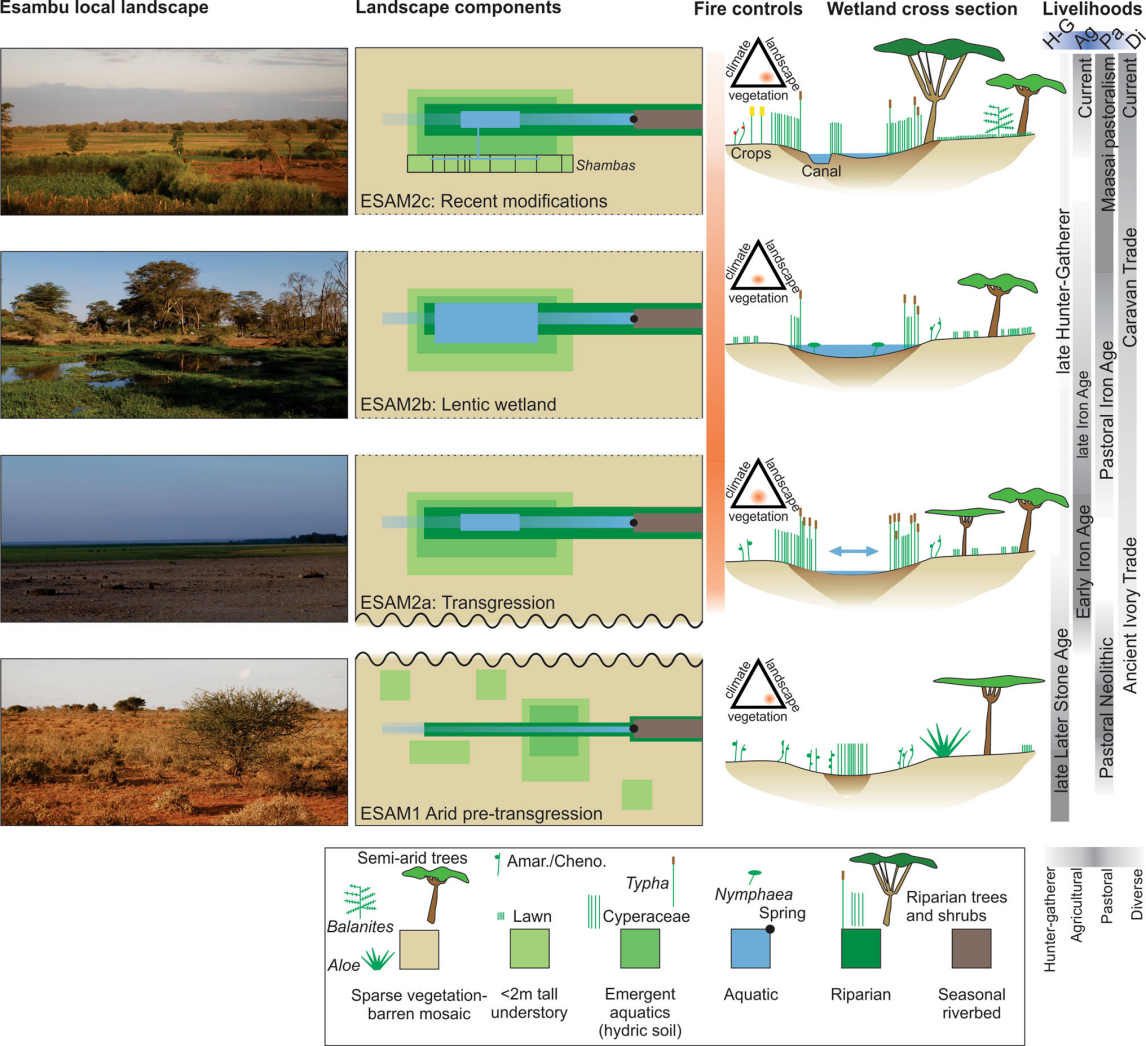
Conceptual interpretation of the ontogeny of Esambu based on pollen and charcoal data and modern history with zonation based on CONISS delineation of the pollen record and with consideration of the age-depth model uncertainties. Note slight rounding of the zone boundary dates. **a** Schematic of the local landscape hydrological and vegetation components with corresponding colours for plant functional types in the legend at bottom. Less abrupt transitions between zones are delimited by dashed line. Components are not to strict scale, although relative size upon the local landscape is suggested. The human developments illustrated in the modern zone primarily apply to contemporary and recent history and are superimposed but not derived from interpretations of the pollen and charcoal data. **b** Fire triangles representing three major sets of variables that are important controls on fire regimes at local-to-landscape scales and decadal-to-millennial temporal scale. **c** A cross-sectional diagram of Esambu Swamp and the major vegetation and landscape changes interpreted from the sediment record. Vegetation types and inferred structure are depicted by the plant types and distributions that correspond to the landscape components in the legend below. **d** Summary of the continuum of livelihood traditions and material cultures that were potentially important to the varying and different anthropogenic impacts on the landscape (Foley 1981; Lane 2013)

The pollen, NPP and charcoal data, combined with contemporary observations on the wetlands, were used to produce a schematic model of the landscape transitions (Fig. 6). The earliest part of the record, around 5000 cal year BP, can be summarised as a predominantly semi-arid landscape with woodland and scrub taxa with limited fuel connectivity and a relatively constrained Cyperaceae dominated wetland. There followed a period of increased moisture that resulted in the wetland expansion increased occurrences of standing water and Cyperaceae and *Typha* growth about the wetland margin. Within the catchment, there was increased woody vegetation, fuel connectivity and biomass burning. The uppermost part of the sequence records intensification of human modifications to the wetland area for agriculture; changing land use around Esambu swamp has led to the conversion of the *Typha*-dominated swamp into agricultural production (*shambas* and commercial farming) as marked by the influx of crops and ruderal taxa. Biomass burning is very low, principally due to the lack of fuel (potentially increased grazing intensity) within the catchment and this is what the landscape currently looks like.

## Discussion

### Ecosystem and fire history of the Esambu catchment

The Esambu wetland site is representative of local (≤ 10 km^2^) vegetation shifts due to its small size (~ 0.4 km^2^). In addition to this local palaeoenvironmental signal, some Afromontane pollen grains were present, mostly likely derived from the surrounding Chyulu Hills and Kilimanjaro that are indicative of more regional ecosystem changes. A combination of climatic changes, human-herbivore interactions and fires are the key drivers that would have caused the ecosystem to change in composition and distribution. Fires are an important controlling factor within savanna ecosystems and occur regularly with their impact largely controlled by the interaction between climate variability, rates of primary productivity, distribution of fuels, and human land use (Colombaroli et al. 2014). Due to uncertainties with the chronology in the lower part of the core, the ecosystem changes will be presented in three broad periods relating to the mid Holocene, the last two millennia and the last four to five centuries.

From ~ 5000 cal year BP through mid-Holocene, the ecosystem was open patchy savanna dominated by shrubs and trees mainly Amaranthaceae/Chenopodiaceae and *Acacia* (Fig. 5). Fuels from shrubs and herbs within the Amaranthaceae/Chenopodiaceae landscape are less continuous than more mesic savannas, which support continuous Poaceae-dominated swathes, and are characterised by low-intensity fires (Murphy et al. 2013). Low levels of biomass burning are apparent in the period covered by the mid Holocene Esambu sediments, reflective of the local dry landscape and the pollen record reveals low abundances of herbaceous vegetation and lack of continuous understory. This late Holocene savanna ecosystem was characterised by low fuel connectivity; lack of continuous understory vegetation and abundant woody species means increased areas of open space that act as firebreaks for ground fires.

From the late Holocene to c. 500–400 cal year BP, there is an increase in pollen diversity as the ecosystem surrounding Esambu Swamp became more mesic with an increase in woodland (*Commiphora*-dominated) and shrub/grassland taxa. Afromontane forest on the adjacent highlands also expanded during this time (Figs. 5 and S2). This local and regional increase in arboreal cover is likely to result from humid and warm conditions manifest as an expansion of Afromontane forest to low altitudes on Kilimanjaro. The period towards c 400 cal year BP has higher charcoal accumulation rates, accompanied by an increase in Afromontane, woody and understory taxa, which we interpret as resulting from increased moisture availability that increased fuel connectivity, and thus increased local burning. Changing pyrodiversity (variation in the overall fire regime) is an important process in grass-dominated ecosystems in Africa, potentially promoting animal and bird richness in mesic savanna areas (Hempson et al. 2017). This signal of increased burning from Amboseli may have been coeval with increased biomass burning reported from the Laikipia Plateau of central Kenya c. 1900–1700 cal year BP (Taylor et al. 2005); this could represent a more regional shift possibly driven by higher precipitation and ensuing biomass cover and fuel connectivity for burning. High spore abundance with *Chaetomium*, *Coniochaeta* cf. *Ligniaria*, *Sporormiella*, T. HdV-1020 and cf. *Valsaria* sp. dominating are indicators of high herbivore density (van Geel et al. 2011), as herbivores were concentrated within a relatively small area to access water and grazing from c.800 to 350 cal year BP. This indicates that herbivore densities were high prior to the extreme defaunation that took place during intense ivory trading of the last millennium (Håkansson 2004) driven by European, American and more recently Asian exogenous markets.

After c. 400 cal year BP, when the chronology becomes more secure, the number of Afromontane and woody-shrubland taxa declined. This could have been as a result of human modifications to forest compositions on the mountain slopes, which were cleared in patches and transformed into the mixed agroforestry systems typified by the *Chagga* home gardens on Kilimanjaro (Hemp 2005) and the Pare Mountains (Finch et al. 2016) of northern Tanzania. This period of marked intensive forest clearance and expansion of sedentary agriculture in the wider region is quite transformative and tied into caravan trades, largely for ivory, and the expansion of New World crop imports such as maize, potatoes and tomatoes. Through this period, there is evidence of highly variable environmental conditions within the Esambu catchment as *Typha* and *Nymphaea* decreased and increased; the latter a signal of open water conditions from 400 to 300 cal year BP followed by a drying episode (Fig. 5). Severe droughts lasting decades across equatorial eastern Africa have been observed in historical and sedimentary records (Rourke 2011). The coprophilous spore taxa (*Sporormiella*) are still consistently present (Fig. 5) suggesting little change in the overall large herbivore density population. However, the source of the dung could have been domesticated species of ungulates (van Geel et al. 2011). Between 400 and 300 cal year BP, mean CHAR values remained low until present, probably because of less plant biomass in the local ecosystem. Current populations near Chyulu Hills apply fire to vegetation cover between May and October (Kamau and Medley 2014) and MODIS satellite observations show relatively few fires in the Esambu area (Fig. 1 inset). The relationship between *Sporormiella* and Cyperaceae suggests that the density of herbivores at the coring location was higher when Cyperaceae relative abundance increased and aquatic pollen diversity decreased when water level was lower. Aquatic taxa were less abundant but Cyperaceae abundances were maintained by growing in the hydric soils of the moist wetland margin. Currently fires in the Esambu swamp catchment result following human-induced ignition and the spread is driven by limited by fuel abundances, conditions and connectivity. Barren ground patches such as those seen in Fig. 3a limit the spread of fire in many ecosystems (Colombaroli et al. 2014) and grazing intensities can reduce fuel connectivity leading to short-stature lawns, especially during the dry seasons when conditions are more conducive to burning (Archibald et al. 2005).

### Disentangling drivers of ecosystem change: insights for management

East African savanna palaeoenvironmental records indicate a highly variable environment from the mid Holocene to present environment (Thompson et al. 2002; Gillson 2004; Rucina et al. 2010). The Esambu pollen record is typical of a semi-arid savanna pollen record with the common savanna trees, grasses, local aquatic taxa combined with the deposition of well-dispersed montane taxa. Around 5000 cal year BP different sites across East Africa showed an increase in abundance of Poaceae at the expense of arboreal taxa in the lowlands, coupled with increases in montane forest taxa indicative of drier climatic conditions. The East African environment shifted towards a drier climate regime characterised by reduced precipitation, increased evaporation, and/or an extension or intensification of the dry season (Marchant and Hooghiemstra 2004). Isotopic data at Lake Malawi show an increase in C_4_ vegetation, initiated again around 4000 cal year BP, which is indicative of increasingly arid environmental conditions (Castañeda et al. 2009). A peak in aeolian dust around 4000 cal year BP also indicates regionally dry conditions resulting in increased dust deposition to the Kilimanjaro glacier (Thompson et al. 2002).

The Esambu record is characterised by a highly diverse mix of savanna and montane taxa, most likely a local signal of increased moisture in the mid to late Holocene (200–131 cm). A record from the Amboseli Basin at Namelok Swamp records a relatively moist environment about this time with the increased presence of *Syzygium*, a tree taxon that grows within waterlogged soils (Rucina et al. 2009) between 2150 and 800 cal year BP. After ~ 800 cal year BP, the record from Namelok Swamp indicates a drier environment with the increased presence of Amaranthaceae and Poaceae and decreased levels of *Syzygium* (Rucina et al. 2009). A dry climate, with increasing signals of human-ecosystem interaction, is observed from Lakes Naivasha and Challa (Westerberg et al. 2010; Buckles et al. 2015). Within the last c. 400 years (≤ 131 cm), the Esambu record was characterised by a decrease in abundance and diversity of woody taxa and an increase in the Cyperaceae-Poaceae ratio that could have resulted from increased human activity that impacted on the surrounding forests. The accompanying increase in charcoal abundance might have resulted from increased natural and anthropogenic ignition coupled with increased fuel connectivity and potentially lower grazing pressures.

Humans have been present within the landscape for many millennia: archaeological studies from Tsavo show evidence of domesticates from faunal diagnostic finds identifying the presence of cattle *Bos taurus* (Wright et al. 2007) from 3700 cal year BP. Despite this longevity of human presence, it is only fairly recently that human agency has become one of the key drivers of ecosystem composition and distribution. Pollen evidence for intensive human-induced land use modifications to local forest ecosystems extend back to c. 600 cal year BP at on Mount Shengena, northern Tanzania (Finch et al. 2016). Sustained and pervasive human impact, in the form of intensive crop cultivation across East Africa, occurs a little later, particularly over the last three to four centuries. Expanding forest clearance, particularly on the highlands of East Africa (Finch et al. 2016) and expansion of sedentary agriculture in the wider region is quite transformative on human-ecosystem interactions and tied into external drivers, particularly the expanding and increasing industrial ivory caravan routes and the arrival of New World crops. *Zea mays* can be detected within sedimentary records dates to 1810 AD on the Pare Mountains (Finch et al. 2016) and around 1650 AD at Lake Naivasha (Lamb et al. 2003). At Esambu, the presence of Poaceae pollen grains > 60 µm, which can be interpreted as being derived from cereal grasses, are accompanied by increased presence of macroscopic charcoal, and thought to be derived from more localised increased farming activity. Increased presence of *Acacia*, Amaranthaceae/Chenopodaceae, *Balanites* and Poaceae in the uppermost samples from Esambu, dating to the last 200 years, could be indicative of a drier environment, or a consequence of a decreased elephant population. Herbivores and elephants in particular, impart a major influence on vegetation composition, distribution and structure (Håkansson 2004; Guldemond and Van Aarde 2008). Although largely unquantified, the removal of millions of rhinoceros and elephants from the East African landscape via the ivory trade, that reached industrial proportions in the 18th and 19th centuries, would have been transformative on savanna and woodland composition and distribution.

Thus, there have been a range of ecosystem shifts within the Amboseli landscape that have increasingly moved from being driven by environmental variability towards anthropocentric factors, either via impact on elephant population, import of new crops or expansion of sedentary agriculture in replacement of nomadic pastoralism. These long-term insights into ecosystem human–environment interactions can be vital to inform adaptive management policies and defining boundary condition that such policies may want to achieve (Gillson and Marchant 2014). Such long-term insights are particularly important under current human-induced land use transformations and increasing impact of climate change and climate variability on the changing distribution of species (Platts et al. 2012). However, for these data to be able to inform such debates they have to be communicated to a range of stakeholders and the presentation of complex stratigraphic data (Figs. 4, 5) as a simple illustration (Fig. 6) is one potential way to make these data more accessible to a wider audience and can be incorporated into video media and made available on different platforms.

Although the palaeoecological study presented from Esambu swamp does not present a ‘pre-disturbance baseline’ to provide a management target, for example via for restoration ecology interventions to work towards some pristine natural state, it does provide an understanding of the local response of such wetlands to the regional drivers of change. Such understanding is crucial as these wetlands, and indeed the wider hydrological budget, is under increasing pressure through a combination of changing climates, population growth, sedentarisation and land use policies in a complex institutional landscape. Such insights can support the development of customised management plans that consider these interacting factors in operation across the landscape and clearly the influence of the different factors change through time. Viewing the Amboseli landscape as one that has been, and continues to be, transformed through centuries of human agency, provides temporal context for understanding the interactions that take place between the wildlife, livestock, people, and their ecosystems.

## Conclusions

The pollen, NPP and charcoal in the Esambu sediment record provide a long-term record of ecosystem dynamics from the Amboseli, albeit with some chronological uncertainties. Sparse woodland and shrub taxa characterised the semi-arid Amboseli landscape from c. 5000 to sometime between 3500 and 500 cal year BP. Wetland expansion resulted in peat accumulation and preserved a high resolution record of vegetation variability in response to hydroclimatic variability and modifications by herbivores and, more recently, human land use changes. Current land cover and land use impacts over the past centuries years have resulted in the conversion of a portion of the wetland to agricultural production as human activity becomes much more visible within the Esambu wetland. Future studies in the region and at comparable wetlands in eastern Africa should focus on sedimentological analyses, relationships between sedimentology and geochronology, in addition to palaeovegetation.

The current Esambu swamp ecosystem has no analogue in the last c. 5000 cal year BP; this indicates the dynamic nature of the ecosystem as current conditions impact on the swamp leading to the emergence of a novel ecosystem reflecting modern day pressures. Understanding how ecosystems have evolved under these changing interactions of climate variability, human land use and land cover, wildlife use, and fire activity through time and across space is vital. This provides an informed context for future management choices that need to take into account that the system is shaped by numerous anthropogenic fingerprints through time.

## Electronic supplementary material

Below is the link to the electronic supplementary material.

Supplementary material 1 (PDF 848 kb)
