# Supplementary material for: Late Holocene wetland transgression and 500 years of vegetation and fire variability in the semi-arid Amboseli landscape, southern Kenya
Source: Ambio. 2018 Feb 3;47(6):682–96. doi: 10.1007/s13280-018-1014-2 (PMC6131128; doi:10.1007/s13280-018-1014-2)
Supplement: Supplementary file 1 — Supplementary material 1 (PDF 848 kb) [file 13280_2018_1014_MOESM1_ESM.pdf]

Ambio

Electronic Supplementary Material

This supplementary material has not been peer reviewed.

Late Holocene wetland transgression and 500 years of vegetation and fire variability in the semi-arid Amboseli landscape, southern Kenya

Article No: s13280-018-1014-2

Esther N Githumbi[1, *], Colin J Courtney Mustaphi[1, 2, *], Kevin J Yun[3], Veronica Muiruri[4, 5], Stephen M Rucina[4], Rob Marchant[1]

[1] York Institute for Tropical Ecosystems, Environment Department, University of York, York, United Kingdom

[2] Department of Archaeology and Ancient History, Uppsala Universitet, Uppsala, Sweden

[3] School of Biological and Biomedical Sciences, Durham University, Durham, United Kingdom

[4] Department of Earth Sciences, Palynology and Palaeobotany Section, National Museums of Kenya, Nairobi, Kenya

[5] Hong Kong Baptist University, Hong Kong, China

* Authors contributed equally

Corresponding author:

Esther Githumbi, York Institute for Tropical Ecosystems, Environment Department, University of York, Heslington, York, YO10 5NG, United Kingdom

Email: esther.githumbi@york.ac.uk

SUPPLEMENTARY MATERIAL

Figure S1: Wood fragment found at 180.5-186 cm depth in the Esambu sediment core that was radiocarbon dated to 394±24 ^{14}C years BP, $F^{14}C$ 0.9522 ± 0.0029 (UBA-26124) and identified as African acacia (*Vachellia* sp.). Prepared wood thin sections were examined through optical photomicroscopy of wood porosity (disposition, size), disposition of parenchyma and fibre of transversal thin section cuts by Delphine Joly and Robyn Veal. Photograph at left shows a fragment of the wood embedded in plastic resin at the Department of Archaeology, University of York, York, UK, by Dr. Carol Lang. Transversal thin sections at 50x (middle photograph) and 100x magnification in optical light (right photograph).

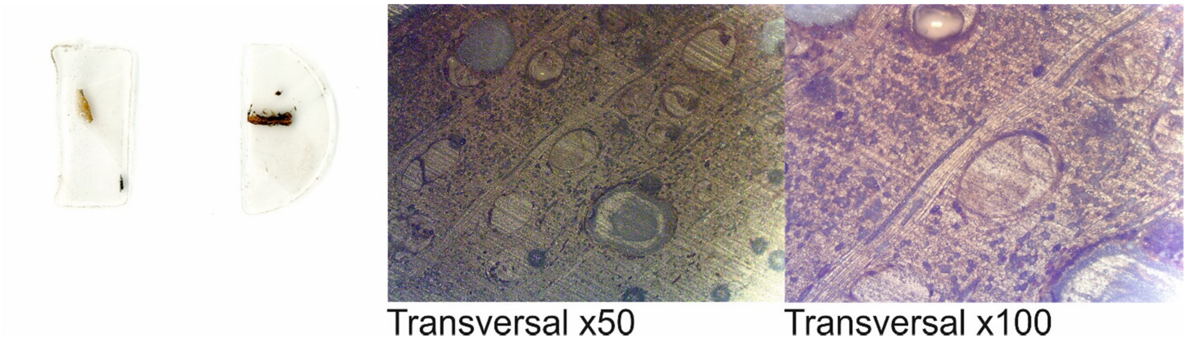

Figure S1

Figure S2: Selected taxa from the Esambu sediment record and published regional records of the past 500 years. Charcoal shown as CHAR (charcoal accumulation rate, pieces cm^{-2} yr^{-1}) Other data shown are the first principal component (PCA; Tierney et al., 2013) of three hydroclimatic proxy records (Verschuren et al., 2009; Tierney et al., 2011; Wolff et al., 2011; Buckles et al., 2015) from Lake Challa and a lake level reconstruction from Lake Naivasha (Verschuren et al., 2000). Reconstructed crop and pasture land cover extracted from the HYDE 3.1 land-cover database covering East Africa (Klein Goldewijk et al., 2010; 2011).

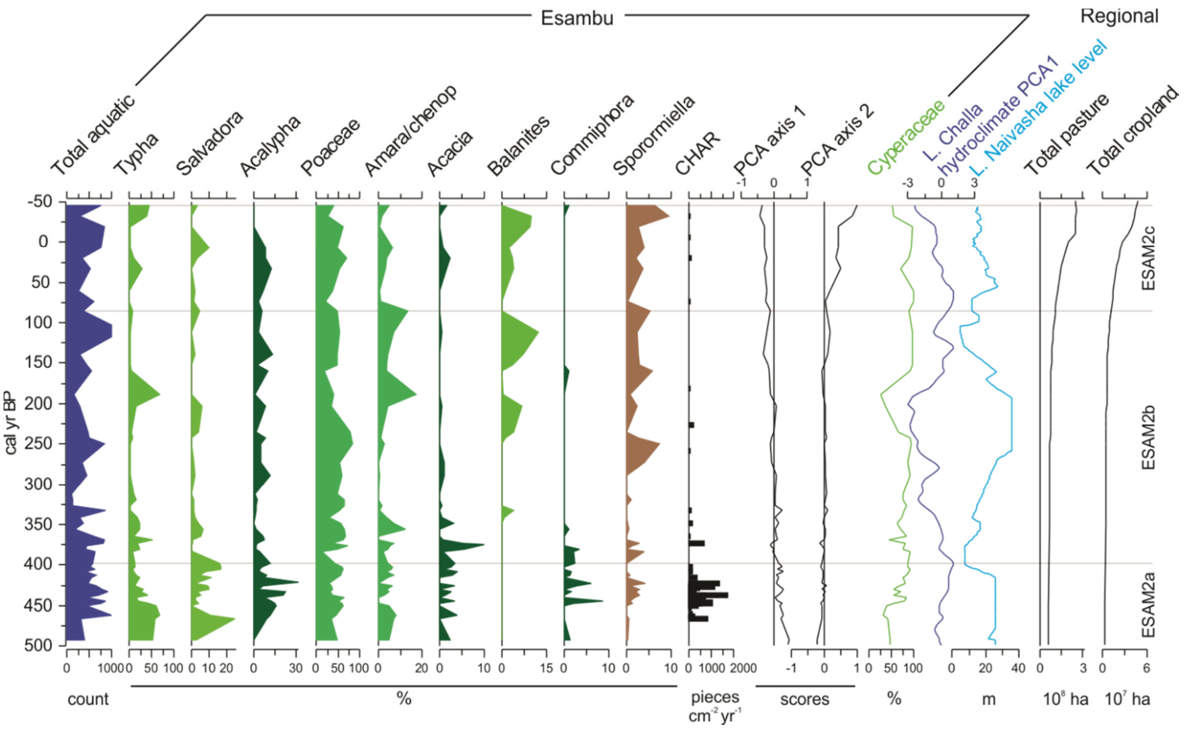

Figure S2

Table S1: Pollen taxa identified the biomes they occupy and the plant structure. The pollen identified is placed into its genera and family classification, an “x” highlights the biomes the taxa occupies and the plant structure. UMF=Upper Montane Forest, M-LMF=Middle and Lower Montane Forest.

		Biome							Structure				
**Family**	**Genera**	**UMF >2500m**	**M-LMF <2500m**	**Woodland**	**Savannah**	**Shrubland**	**Riverine**	**Aquatic**	**Trees**	**Shrubs**	**Forbs**	**Grasses**	**Aquatic**
Aquifoliaceae	Ilex	X	X						X	X			
Araliaceae	Hydrocotyle							X					X
Araliaceae	Schefflera		X	X					X	X			
Boraginaceae	Cordia	X	X	X	X				X				
Brassicaceae	Brassicaceae		X	X	X	X					X		
Burseraceae	Commiphora		X	X	X	X			X	X			
Cannabaceae	Celtis	X	X						X				
Capparaceae	Capparis		X	X	X	X				X	X		
Combretaceae	Terminalia		X	X	X	X			X				
Cupressaceae	Juniperus	X	X						X				
Cyperaceae	Cyperus						X	X				X	X
Ebenaceae:	Euclea	X	X	X	X	X							
Euphorbiaceae	Croton	X	X	X					X				
Euphorbiaceae	Euphorbia	X	X	X	X	X	X			X	X		
Fabaceae	Tamarindus		X	X	X	X			X				
Haloragaceae	Myriophyllum						X	X					X
Loganiaceae	Nuxia	X	X						X				
Mimosaceae	Acacia		X	X	X	X	X		X	X			
Moraceae	Ficus	X	X	X	X	X	X		X	X	X		
Moraceae	Trilepsium	X	X	X	X	X	X		X				

Nymphaeaceae	Nymphaea							X					X
Oleaceae	Olea	X	X						X				
Pinaceae	Pinus	X	X						X				
Poaceae	Poaceae	X	X	X	X	X	X	X			X	X	
Podocarpaceae	Podocarpus	X	X						X				
Primulaceae	Rapanea	X	X						X				
Rosaceae	Hagenia	X	X						X				
Rubiaceae	Rubiaceae	X	X	X	X				X	X	X	X	
Typhaceae	Typha												
Zygophyllaceae	Balanites						X	X	X	X			X
